# Some Notes on Maximum Entropy Utility

**DOI:** 10.3390/e21070637

**Published:** 2019-06-27

**Authors:** Eun Young Kim, Byeong Seok Ahn

**Affiliations:** 1 Department of Neurosurgery, Gachon University Gil Medical Center, 21 Namdongdaero 774, Namdong, Incheon 21565, Korea; 2 College of Business and Economics, Chung-Ang University, 221 Heukseok Dongjak, Seoul 06974, Korea

**Keywords:** decision analysis, utility, maximum entropy

## Abstract

The maximum entropy principle is effective in solving decision problems, especially when it is not possible to obtain sufficient information to induce a decision. Among others, the concept of maximum entropy is successfully used to obtain the maximum entropy utility which assigns cardinal utilities to ordered prospects (consequences). In some cases, however, the maximum entropy principle fails to produce a satisfactory result representing a set of partial preferences properly. Such a case occurs when incorporating ordered utility increments or uncertain probability to the well-known maximum entropy formulation. To overcome such a shortcoming, we propose a distance-based solution, so-called the centralized utility increments which are obtained by minimizing the expected quadratic distance to the set of vertices that varies upon partial preferences. Therefore, the proposed method seeks to determine utility increments that are adjusted to the center of the vertices. Other partial preferences about the prospects and their corresponding centralized utility increments are derived and compared to the maximum entropy utility.

## 1. Introduction

The maximum entropy principle is effective in solving decision problems, especially when it is not possible to obtain sufficient information to induce a decision [1,2,3,4,5]. Applications of the entropy principle to the multiple criteria decision-making (MCDM) problems can be found in [6,7,8]. Abbas [9] presents a method to assign cardinal utilities to ordered prospects (consequences) in the presence of uncertainty. The ordered prospects which are included in the category of partial preferences are easily encountered in practice [9,10,11]. The use of partial preferences about the prospects can provide a decision-maker with comfort in specifying preferences but in view of decision-making may fail to result in a final decision. Thus, an elegant approach to circumvent this problem is needed to solve real-world decision-making problems. To this end, Abbas [9] developed the maximum entropy approach to assigning cardinal utility to each prospect when only the ordered prospects are known. However, we doubt if the maximum entropy approach results in cardinal utilities representing a set of partial preferences properly where some other partial preferences about the prospects are additionally incorporated. In another context of true maximum ignorance where the state of prior knowledge is not strong, the maximum a posteriori probability can be better estimated by classical Bayesian theory; it is not necessary to introduce a new and exotic approach such as maximum entropy [12].

We discuss the maximum entropy utility approach further using the notations and definitions from Abbas [9].

## 2. Does the Maximum Entropy Principle Always Guarantee a Good Solution?

A utility vector contains the utility values of prospects starting from the lowest to the highest, where a utility value of zero (one) is assigned to the lowest (highest) according to a von Neumann and Morgenstern type utility assessment. We assume that there is at least one prospect, which has a strict preference to exclude the case of absolute indifference. The utility vector for (K+1) prospects can be denoted by

$$\begin{array}{cc} U & ≜\left(U_{0},\;U_{1},\cdots ,U_{K-1},\;U_{K}\right)\\ & =\left(0,\;U_{1},\;U_{2},\cdots ,U_{K-1},\;1\right) \end{array}$$

where $0\leq U_{1}\leq U_{2}\leq \cdots \leq U_{K-1}\leq 1$.

A utility increment vector ΔU, whose elements are equal to the difference between the consecutive elements in the utility vector, can be denoted by

$$\Delta U≜\left(U_{1}-0,\;U_{2}-U_{1},\cdots ,1-U_{K-1}\right)=\left(\Delta u_{1},\;\Delta u_{2},\Delta u_{3},\cdots ,\Delta u_{K}\right).$$

A utility increment vector ΔU satisfies two properties: (1) $\Delta u_{i}\geq 0$, i=1,⋯,K and (2) $\sum_{i=1}^{K}\Delta u_{i}=1$. Thus, it represents a point in a K-dimensional simplex, so-called the utility simplex. To assign cardinal utility to each $\Delta u_{i}$, Abbas [9] presumed that “If all we know about the prospects is their ordering, it is reasonable to assume, therefore, that the location of the utility increment vector is uniformly distributed over the utility simplex.” This idea led to the following nonlinear program (Equation (1)) of which the objective function is the maximum entropy constrained by a normalization condition and non-negativity constraints:

(1) $$\begin{array}{ll} \Delta U_{maxent}= & \underset{\Delta u_{1},\;\Delta u_{2},\cdots ,\;\Delta u_{K}\;}{\mathrm{maximize}}-\sum_{i=1}^{K}\Delta u_{i}\log\left(\Delta u_{i}\right)\\ \text{such that} & \\ & \sum_{i=1}^{K}\Delta u_{i}=1\\ & \Delta u_{i}\geq 0,\;i=1,\cdots ,K. \end{array}$$

The optimal solution to this program is a utility increment vector with equal increments, that is

(2) $$\Delta u_{i}=\frac{1}{K},\;i=1,\cdots ,K.$$

This result seems to properly represent the utility simplex, since its extreme points simply consist of K unit vectors $e_{i}$ (one in the *i*th element and zeroes elsewhere) of which the coordinate-wise average yields $\frac{1}{K}$. 

Let us assume an ordered increasing utility (OIU) increment (in the latter part of the paper, we provide the ordered decreasing utility (ODU) increment defined by $\Delta u_{1}\geq \Delta u_{2}\geq \cdots \geq \Delta u_{K}$):

(3) $$\Delta u_{1}\leq \Delta u_{2}\leq \cdots \leq \Delta u_{K},$$

which can be further rewritten as $U_{1}-0\leq U_{2}-U_{1}\;\leq \cdots \leq 1-U_{K-1}$ in terms of the utility vector. Studies regarding this partial preference, also called comparable preference differences, degree of preference, strength of preference, or preference intensity to utility theory, are found in Fishburn [13] and Sarin [14].

**Figure 1:**
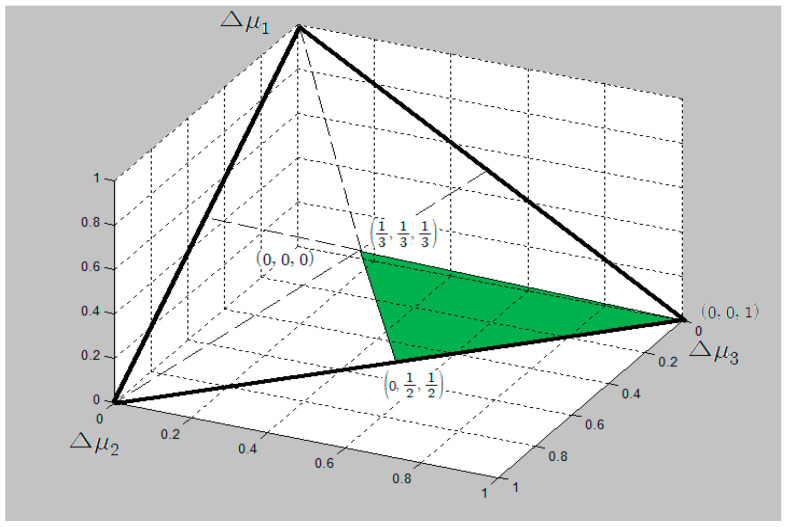
A utility simplex constrained by an ordered increasing utility increment.

The incorporation of the ordered utility increment vector in the system of constraints of Equation (1) leads to the mathematical program (Equation (4)) and surely restricts the utility simplex as depicted in Figure 1, when considering a case of K=3.

(4) $$\begin{array}{ll} \Delta U_{maxent}= & \underset{\Delta u_{1},\;\Delta u_{2},\cdots ,\;\Delta u_{K}\;}{\mathrm{maximize}}-\sum_{i=1}^{K}\Delta u_{i}\log\left(\Delta u_{i}\right)\\ \text{such that} & \\ & \Delta u_{1}\leq \Delta u_{2}\leq \cdots \leq \Delta u_{K}\\ & \sum_{i=1}^{K}\Delta u_{i}=1\\ & \Delta u_{i}\geq 0,\;i=1,\cdots ,K. \end{array}$$

The solution to Equation (4) however still yields a utility increment vector with equal increments, $\Delta u_{i}=\frac{1}{K}$, i=1,⋯,K since nothing other than $v_{K}$ can result in a larger maximum entropy in a set of extreme points $\left\{v_{1},v_{2},\cdots ,v_{K}\right\}$ where $v_{1}=\left(0,\;0,\cdots 0,\;0,\;1\right)$, $v_{2}=\left(0,\;0,\cdots ,0,\;\frac{1}{2},\;\frac{1}{2}\right)$, ⋯, $v_{K}=\left(\frac{1}{K},\frac{1}{K},\cdots \frac{1}{K},\;\frac{1}{K}\right)$.

**Figure 2:**
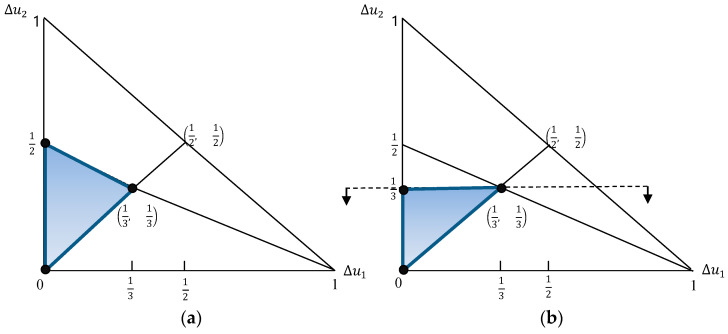
Utility simplex with additional constraints in case of K=3. (**a**) $\Delta U_{1}=\left\{\Delta u:\;\Delta u_{3}\geq \Delta u_{2}\geq \Delta u_{1},\;\sum_{i=1}^{3}\Delta u_{i}=1\right\}$ (**b**) $\Delta U_{2}=\Delta U_{1}\cap \left\{\Delta u:\;\Delta u_{3}-\Delta u_{2}\geq \Delta u_{2}-\Delta u_{1},\;\sum_{i=1}^{3}\Delta u_{i}=1\right\}$.

Technically, we always obtain this result when the constituent constraints in the maximum entropy program contain $v_{K}$ as one of their extreme points. To illustrate, let us incorporate a constraint $\Delta u_{3}-\Delta u_{2}\geq \Delta u_{2}-\Delta u_{1}$ which more restricts the utility simplex in Figure 1 (see Figure 2). The set of extreme points is composed of $v_{1}=\left(0,\;0,\;1\right)$, $v_{2}=\left(0,\;\frac{1}{3},\;\frac{2}{3}\right)$, $v_{3}=\left(\frac{1}{3},\;\frac{1}{3},\;\frac{1}{3}\right)$, which also leads to a maximum entropy merely anchored at $v_{3}$.

Therefore, it is doubtable if such equal utility increments adequately represent the feasible region (i.e., the restricted utility simplex) and if the assignment of such values will eventually be valid. 

Let us consider another case where the maximum entropy principle does not work properly. We present the discrete version of preference inclusion in the maximum entropy utility, originally dealt with by Abbas [9] in a continuous case. Let us assume that a decision-maker specifies indifference between a lottery $\langle x_{1},\;p_{1};x_{2},\;p_{2};\cdots ;x_{K},\;p_{K}\rangle$ and a reference lottery $\langle x_{K},\;p,\;x_{1}\rangle$, thus yielding

(5) $$\sum_{i=1}^{K}p_{i}U_{i}\left(x_{i}\right)=p\;\mathrm{where}\;x_{1}\leq \;x_{2}\leq \cdots \leq x_{K}.$$

Equation (5) can be rewritten in terms of the utility increments $\Delta u_{i}$ such that $\sum_{i=1}^{K}F_{i-1}\Delta u_{i}=1-p$ where $F_{i}=\sum_{j=1}^{i}p_{j}$ ($F_{0}=0$, $F_{K}=1)$. 

Then, the principle of maximum entropy utility leads to the following program:

(6) $$\begin{array}{ll} \Delta U_{maxent}= & \underset{\Delta u_{1},\;\Delta u_{2},\cdots ,\;\Delta u_{K}\;}{\mathrm{maximize}}-\sum_{i=1}^{K}\Delta u_{i}\log\left(\Delta u_{i}\right)\\ \text{such that} & \\ & \sum_{i=1}^{K}F_{i-1}\Delta u_{i}=1-p\\ & \sum_{i=1}^{K}\Delta u_{i}=1\\ & \Delta u_{i}\geq 0,\;i=1,\cdots ,K. \end{array}$$

The solution to Equation (6) is obtained by

(7) $$\Delta u_{i}=\frac{\exp\left(\beta F_{i-1}\right)}{\sum_{i=1}^{K}\exp(\beta F_{i-1})}$$

where β corresponds to the Lagrange multiplier and is determined iteratively from the equation $\sum_{i=1}^{K}\left(F_{i-1}-\left(1-p\right)\right)\exp(\beta F_{i-1})=0$. A formulation similar to Equation (6) and its solution (Equation (7)) are found in different contexts [15,16,17]. If a decision-maker is uncertain about the probability that equates a discrete lottery with a reference lottery and thus specifies a probability interval $\underline{p}\leq \tilde{p}\leq \bar{p}$ as in [18], the expected utility of the prospects in Equation (5) can be expressed in the form of an interval:

(8) $$\underline{p}\leq \sum_{i=1}^{K}p_{i}U_{i}\left(x_{i}\right)\leq \bar{p},\;\mathrm{or}\;1-\bar{p}\leq \sum_{i=1}^{K}F_{i-1}\Delta u_{i}\leq 1-\underline{p}.$$

With Equation (8) added in Equation (1), we obtain the optimal utility increments vector to Equation (9) that is anchored at either the lower or the upper bound in Equation (8).

(9) $$\begin{array}{ll} \Delta U_{maxent}= & \underset{\Delta u_{1},\;\Delta u_{2},\cdots ,\;\Delta u_{K}\;}{\max}-\sum_{i=1}^{K}\Delta u_{i}\log\left(\Delta u_{i}\right)\\ \text{such that} & \\ & 1-\bar{p}\leq \sum_{i=1}^{K}F_{i-1}\Delta u_{i}\leq 1-\underline{p}\\ & \sum_{i=1}^{K}\Delta u_{i}=1\\ & \Delta u_{i}\geq 0,\;i=1,\cdots ,K. \end{array}$$

For example, let K=5, $F_{i}=\frac{i}{5}$, i=1,⋯,5, and $\tilde{p}\in \left[0.6,\;0.7\right]$. Then, if we simply let $\Delta u_{i}=\frac{1}{5}$ for all i, we obtain the maximum entropy value while they satisfy all the constraints in Equation (9), that is, $\sum_{i=1}^{5}\frac{F_{i-1}}{5}=0.4=1-\underline{p}$ and $\sum_{i=1}^{5}\frac{1}{5}=1$. Rather than this optimal solution, however, it is more reasonable to expect to obtain utility increments corresponding to somewhere between $\Delta u_{i}\left(0.6\right)$ and $\Delta u_{i}\left(0.7\right)$ in Equation (7). Further, this undesirable result is observed while uncertain $\tilde{p}$ varies upon [0.6, 0.6+α] or [0.6−α, 0.6], α>0.

## 3. Centralized Utility Increments 

We have shown two examples in which the maximum entropy principle works improperly when the utility simplex is restricted by additional partial preferences. This undesirable outcome can be attributed to the fact that the maximum entropy value is always attained when the equal utility increments vector is one of the extreme points characterizing the restricted utility simplex. Clearly, the utility increments representative of the restricted utility simplex are more likely to be found by considering as many extreme points as possible. Toward this end, we propose new utility increments that minimize the sum of the squared distances from all the extreme points (MSDE) to physically locate the utility increments at the center of the restricted utility simplex. Specifically, the MSDE approach considers the utility increments that minimize the expected quadratic distance to the set of vertices that varies upon types of partial preferences. This leads to the MSDE program in Equation (10):

(10) $$\begin{array}{ll} \mathrm{minimize} & \sum_{i=1}^{K}\sum_{j=1}^{M}{\left(\Delta u_{i}-v_{ij}\right)}^{2}\\ \text{such that} & \\ & \sum_{i=1}^{K}\Delta u_{i}=1\\ & \Delta u_{i}\geq 0,\;i=1,\cdots ,K \end{array}$$

where $v_{ij}$ is the ith entry of the jth extreme point for the ordered prospects and M is the number of extreme points. 

The solution to Equation (10) yields

(11) $$\Delta u_{i}=\frac{1}{M}\sum_{j=1}^{M}v_{ij}=\frac{1}{K}$$

since M=K and $v_{j}=e_{j}$ for all j.

This result is identical to Equation (2), which is compatible with the maximum entropy utility under the utility simplex. If we add the ordered utility increment vector (Equation (3)) to the system of constraints in Equation (10), the MSDE yields a solution:

(12) $$\Delta u_{i}=\frac{1}{K}\sum_{j=K-i+1}^{K}\frac{1}{j},\;i=1,\cdots ,K$$

since $v_{1}=\left(0,\cdots ,0,1\right)$, $v_{2}=\left(0,\cdots ,0,\frac{1}{2},\frac{1}{2}\right)$, ⋯, $v_{K}=\left(\frac{1}{K},\cdots ,\frac{1}{K},\frac{1}{K}\right)$.

This solution, so-called the *centralized utility increments*, is quite different from the equal increments that would have resulted had we solved the program using the maximum entropy principle. In the case of K=3, simply compare $\left(\frac{2}{18},\;\frac{5}{18},\;\frac{11}{18}\right)$ based on the centralized utility increments with $\left(\frac{1}{3},\;\frac{1}{3},\;\frac{1}{3}\right)$ based on the maximum entropy principle.

To show that the solution in Equation (12) truly represents the center of vertices of the restricted utility simplex, we first develop a cumulative discrete utility increments function for $\Delta u_{i}$, $F\left(\frac{i}{K}\right)=\sum_{j=1}^{i}\Delta u_{j}$, i=1,⋯,K and then its continuous function as follows [19]:

(13) $$f_{OIU}\left(x\right)=\{\begin{array}{c} x+\left(1-x\right)\ln\left(1-x\right)\;\;\mathrm{for}\;0\leq x<1\\ 0\;\;\;\;\;\;\;\;\;\;\;\;\;\;\;\;\mathrm{for}\;\;x=1 \end{array}.$$

Similar computations yield a continuous function for ordered decreasing utility increments as follows:

(14) $$f_{ODU}\left(x\right)=\{\begin{array}{c} x\left(1-\ln x\right)\;\;\;\;\mathrm{for}\;0<x\leq 1\\ 0\;\;\;\;\;\;\;\;\;\;\mathrm{for}\;\;x=0 \end{array}.$$

**Figure 3:**
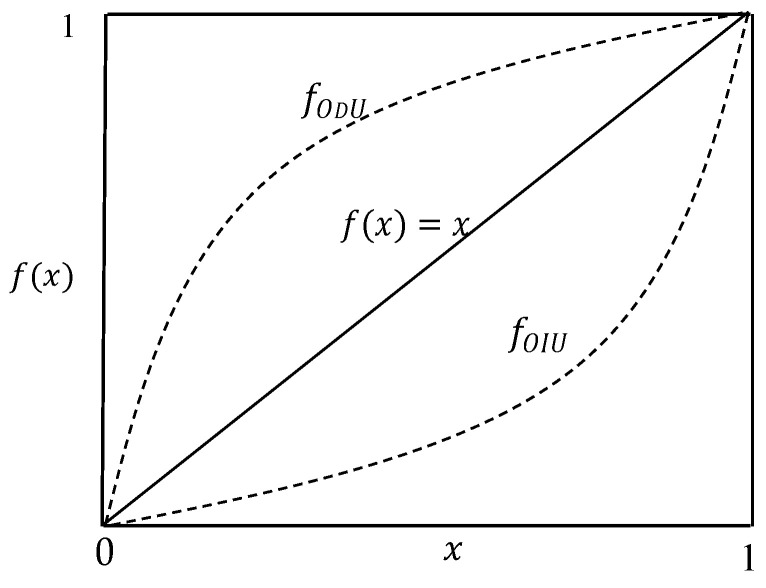
Continuous functions for increasing and decreasing utility increments.

It is interesting to note that both $f_{OIU}\left(x\right)$ and $f_{ODU}\left(x\right)$ include the entropy expression −xlnx as their component. As shown in Figure 3, the continuous functions $f_{OIU}\left(x\right)$ and $f_{ODU}\left(x\right)$ bisect the lower triangle and upper triangle (of an area of $\frac{1}{2}$) respectively since $\int_{0}^{1}f_{OIU}\left(x\right)dx=\frac{1}{4}$ and $\int_{0}^{1}f_{ODU}\left(x\right)dx=\frac{3}{4}$. Noting that the straight line f(x)=x generates equal increments (for $\Delta u_{i}=\frac{1}{K}$, $F\left(\frac{i}{K}\right)=\sum_{j=1}^{i}\Delta u_{j}=\frac{i}{K}$ and $\underset{K\to \infty }{lim}F_{K}\left(x\right)=f\left(x\right)=$) for any K, $f_{OIU}\left(x\right)$ produces the centralized utility increments among numerous continuous functions that generate the utility increments satisfying $\Delta u_{1}\leq \Delta u_{2}\leq \cdots \leq \Delta u_{K}$.

Further, we consider two categories of partial utility values that are widely used in MCDM problems: loose articulation (i.e., open-ended partial preferences of utility values) and interval expressions of utility values. The open-ended partial preferences of utility values may include the following types of preferences (see Ahn [20]):

- Weak preference of utility values (WPU): $U_{WPU}=\left\{U_{i}\geq U_{i-1},\;i=1,\cdots ,K\right\}$
- Strict preference of utility values (SPU): $U_{SPU}=\{U_{i}-U_{i-1}\geq \varepsilon_{i}>0,\;i=1,\cdots ,K\}$
- Weak difference of utility values (DPU): $U_{DPU}=\left\{U_{K}-U_{K-1}\geq \cdots \geq U_{1}-U_{0}\right\}$
- Ratio preference of utility values (RPU): $U_{RPU}=\left\{U_{i}\geq \alpha_{i-1}U_{i-1},\;\alpha_{i-1}\geq 1,\;i=1,\cdots ,K\right\}$.

The interval expressions of utility values may include the following types of preferences:

- Interval utility values (IU): $U_{IU}=\left\{LB_{i}\leq U_{i}\leq UB_{i},\;i=2,\cdots ,K-1\right\}$
- Interval differences of utility values (IDU): $U_{IDU}=\left\{LB_{i}\leq U_{i}-U_{i-1}\leq UB_{i},\;i=1,\cdots ,K\right\}$
- Interval ratios of utility values (IRU): $U_{IRU}=\left\{LB_{i}\leq U_{i}/U_{i-1}\leq UB_{i},i=2,\cdots ,K\right\}$

where $LB_{i}$ and $UB_{i}$ represent the lower and upper bounds, respectively.

Finally, we summarize in Table 1 the formulas of the maximum entropy utility and the centralized utility assignments for the case of the open-ended partial preferences (see more details in Appendix A for types of open-ended partial utility values and Appendix B for types of interval partial utility values respectively).

## 4. Conclusions

We have shown two examples in which the maximum entropy principle fails to produce an outcome representative of partial preferences about prospects. Therefore, we have to be cautious when we rely on the maximum entropy formulation to determine a representative vector over the feasible region of constraints. As an alternative, we propose the centralized utility increments that minimize the sum of squared distances from all the extreme points to physically locate the utility increments at the center of the restricted utility simplex. In particular, discrete and continuous functions are derived to demonstrate better performance of centralized utility increments over maximum entropy utility when the ordered utility increments are incorporated. Further, a range of partial utility values are introduced and their centralized utility assignments are compared with the maximum entropy utilities. However, it should be mentioned that we proposed other partial preferences beyond DPU in an attempt to show how to extend the MSDE approach to other partial preferences, which may not be directly related to the resolution of the problem inherent in the maximum entropy utility approach. 

A final remark is that our proposed approach has the limitation of a deterministic one.

## Figures and Tables

**Table 1:** Partial information about utility values and their centralized utility values.

Partial Utility Value	Maximum Entropy Utility	Centralized Utility Assignment
WPU	$\Delta u_{i}=\frac{1}{K}$	$U_{i}=\frac{i}{K}$ $\Delta u_{i}=\frac{1}{K}$
SPU	$\Delta u_{i}=\frac{1}{K}$ if $\varepsilon_{i}\leq \frac{1}{K}$ for all i $\{\begin{array}{c} \Delta u_{i}=\varepsilon_{i}\;\mathrm{for}\;i\in L=\left\{l:\varepsilon_{l}\geq \frac{1}{K}\right\}\;\;\;\;\;\;\;\;\;\\ \Delta u_{i}=\left(1-\sum_{i\in L}\varepsilon_{i}\right)/\left(K-\left|L\right|\right)\;\;\mathrm{elsewhere} \end{array}$	$U_{i}=\sum_{j=1}^{i}\varepsilon_{j}+\frac{i}{K}\left(1-\sum_{j=1}^{K}\varepsilon_{j}\right)$ $\Delta u_{i}=\varepsilon_{i}+\frac{1}{K}\left(1-\sum_{j=1}^{K}\varepsilon_{j}\right)$
DPU	$\Delta u_{i}=\frac{1}{K}$	$U_{i}=\frac{1}{K}\sum_{j=K-i+1}^{K}\frac{j+i-K}{j}$ $\Delta u_{i}=\frac{1}{K}\sum_{j=K-i+1}^{K}\frac{1}{j}$
RPU	maximize $-\sum_{i=1}^{K}\Delta u_{i}\log\left(\Delta u_{i}\right)$ s.t. $\sum_{j=1}^{i}\Delta u_{j}\geq \alpha_{i-1}\sum_{j=1}^{i-1}\Delta u_{j}$ for all i $\sum_{i=1}^{K}\Delta u_{i}=1$, $\Delta u_{i}\geq 0$	$U_{i}=\frac{i}{K}{\left(\prod_{j=i}^{K-1}\alpha_{j}\right)}^{-1}$ $\Delta u_{i}=\frac{i}{K}{\left(\prod_{j=i}^{K-1}\alpha_{j}\right)}^{-1}-\frac{i-1}{K}{\left(\prod_{j=i-1}^{K-1}\alpha_{j}\right)}^{-1}$

## Appendix A. (Open-Ended Partial Preferences of Utility Values [20])

### Appendix A.1. Strict Preference of Utility Values (SPU): $U_{SPU}=\{U_{i}-U_{i-1}\geq \varepsilon_{i}>0,i=1,\cdots ,K\}$

Let the utility vector $\Delta u_{i}$ denote $\Delta u_{i}=U_{i}-U_{i-1}$, i=1,⋯,K. These substitutions lead to an equivalent set in terms of $\Delta u_{i}$

$$\left\{\Delta u_{i}\geq \varepsilon_{i}>0,\;i=1,\cdots ,K,\;\sum_{i=1}^{K}\Delta u_{i}=1\right\}.$$

Then, we use variable $r_{i}$ to denote $r_{i}=\Delta u_{i}-\varepsilon_{i}$ and $s_{i}$ to denote $s_{i}=r_{i}/\left(1-\sum_{i=1}^{K}\varepsilon_{i}\right)$, which yields $U_{r}$ and $U_{s}$ in sequence:

$$U_{r}=\left\{r_{i}\geq 0,\;i=1,\cdots ,K,\;\sum_{i=1}^{K}r_{i}=1-\sum_{i=1}^{K}\varepsilon_{i}\right\}$$

and

$$U_{s}=\left\{s_{i}\geq 0,\;i=1,\cdots ,K,\;\sum_{i=1}^{K}s_{i}=1\right\}.$$

The extreme points of $U_{s}$ can be easily determined as an identity matrix with a dimension K, and using the identity matrix, we obtain the extreme points of $U_{r}$ as $\left(1-\sum_{i=1}^{K}\varepsilon_{i}\right)e_{i}$, i=1,⋯,K. More computations are required to obtain the extreme points in terms of $\Delta u_{i}$ from $U_{s}$. For example, given $r_{K}=\left(0,\cdots ,\;0,\;1-\sum_{i=1}^{K}\varepsilon_{i}\right)$, we obtain $\left(\varepsilon_{1},\varepsilon_{2},\;\varepsilon_{3},\cdots ,\;\varepsilon_{K-1},\;1-\sum_{i=1}^{K}\varepsilon_{i}+\varepsilon_{K}\right)$ from $\Delta u_{i}=r_{i}+\varepsilon_{i}$, i=1,⋯,K. Continuing in this manner for all $r_{i}$, we obtain a set of extreme points in terms of $\Delta u_{i}$: $\left(1-t+\varepsilon_{1},\;\varepsilon_{2},\cdots ,\;\varepsilon_{K-1},\;\varepsilon_{K}\right)$, $\left(\varepsilon_{1},\;1-t+\varepsilon_{2},\;\varepsilon_{3},\cdots ,\;\varepsilon_{K-1},\;\varepsilon_{K}\right)$, ⋯, $\left(\varepsilon_{1},\varepsilon_{2},\;\varepsilon_{3},\cdots ,\;\varepsilon_{K-1},\;1-t+\varepsilon_{K}\right)$ where $t=\sum_{i=1}^{K}\epsilon_{i}$.

The coordinate averages of these vectors result in the centralized utility assignments $\Delta u_{i}=\varepsilon_{i}+\frac{1}{K}\left(1-\sum_{j=1}^{K}\varepsilon_{j}\right)$ and $U_{i}=\sum_{j=1}^{i}\varepsilon_{j}+\frac{i}{K}\left(1-\sum_{j=1}^{K}\varepsilon_{j}\right)$ from $\Delta u_{i}=U_{i}-U_{i-1}$. If $\varepsilon_{i}=0$ for all i, then a set of strict preference $U_{SPU}$ simply reduces to a set of weak preference $U_{WPU}$ such as $U_{WPU}=\left\{1=U_{K}\geq U_{K-1}\geq \cdots \geq U_{1}\geq U_{0}=0\right\}$.

Therefore, the centralized utility assignments for $U_{WPU}$ prove to be $\Delta u_{i}=\frac{1}{K}$ and $U_{i}=\frac{i}{K}$ from the results of $U_{SPU}$.

### Appendix A.2. Weak Difference of Utility Values (DPU): $U_{DPU}=\left\{U_{K}-U_{K-1}\geq \cdots \geq U_{1}-U_{0}\right\}$

Using equations such that $\Delta u_{i}=U_{i}-U_{i-1}$, i=1,⋯,K leads to a set

$$\left\{\Delta u_{K}\geq \Delta u_{K-1}\geq \cdots \geq \Delta u_{1},\;\sum_{i=1}^{K}\Delta u_{i}=1\right\}$$

and its extreme points are well-known and widely-used in multi-attribute decision analysis with ranked attribute weights [21,22,23,24]: (0, ⋯,0, 1), $\left(0,\cdots 0,\frac{1}{2},\;\frac{1}{2}\right)$, ⋯, $\left(\frac{1}{K},\;\cdots ,\;\frac{1}{K}\right)$. The coordinate averages of these vectors result in the centralized utility assignments $\Delta u_{i}=\frac{1}{K}\sum_{j=K-i+1}^{K}\frac{1}{j}$ and $U_{i}=\frac{1}{K}\sum_{j=K-i+1}^{K}\frac{j+i-K}{j}$.

### Appendix A.3. Ratio Preference of Utility Values (RPU)

$$\mathrm{The}\;\mathrm{ratio}\;\mathrm{preference}\;\mathrm{of}\;\mathrm{utility}\;\mathrm{values},\;U_{RPU}=\{U_{i}\geq \alpha_{i-1}U_{i-1},\;\alpha_{i-1}>0,\;i=1,\cdots ,K\}\;\mathrm{can}\;\mathrm{be}\;$$

rewritten as

$$U_{RPU}=\left\{U_{K}\geq \alpha_{K-1}U_{K-1}\geq \alpha_{K-1}\alpha_{K-2}U_{K-2}\geq \cdots \geq \alpha_{K-1}\cdots \alpha_{1}U_{1}\geq \alpha_{K-1}\cdots \alpha_{0}U_{0}\right\}.$$

The use of variables $r_{i}$ such that

$$r_{i}=\prod_{j=i}^{K-1}\alpha_{j}U_{i}-\prod_{j=i-1}^{K-1}\alpha_{j}U_{i-1},\;i=1,\cdots ,K-1\;\mathrm{and}\;r_{K}=U_{K}-\alpha_{K-1}U_{K-1}$$

leads to a set $U_{r}$:

$$U_{r}=\left\{r_{i}\geq 0,\;i=1,\cdots ,K,\;\sum_{i=1}^{K}r_{i}=1\right\}.$$

Stating from the extreme points $e_{i}$, i=1,⋯,K of $U_{r}$, we solve a set of equations recursively to obtain the extreme points in terms of $U_{i}$. For example, given $e_{1}=\left(1,\;0,\cdots ,\;0\right)$, we obtain $U_{1}=\frac{1}{\alpha_{1}\cdots \alpha_{K-1}}$, $U_{2}=\frac{1}{\alpha_{2}\cdots \alpha_{K-1}}$, ⋯, $U_{K-1}=\frac{1}{\alpha_{K-1}}$, $U_{K}=1$ by solving the following set of equations:

$$r_{1}=\alpha_{K-1}\cdots \alpha_{1}U_{1}-\alpha_{K-1}\cdots \alpha_{0}U_{0}=1,$$

$$r_{2}=\alpha_{K-2}\cdots \alpha_{2}U_{2}-\alpha_{K-1}\cdots \alpha_{1}U_{1}=0,$$

⋯

$$r_{K-1}=\alpha_{K-1}U_{K-1}-\alpha_{K-1}\alpha_{K-2}U_{K-2}=0,$$

$$r_{K}=U_{K}-\alpha_{K-1}U_{K-1}=0.$$

Continuing in this manner for all $e_{i}$, we obtain a set of extreme points represented in terms of $U_{i}$: $\left(\frac{1}{\alpha_{1}\cdots \alpha_{K-1}},\;\frac{1}{\alpha_{2}\cdots \alpha_{K-1}},\;\cdots ,\frac{1}{\alpha_{K-1}},\;1\right)$, $\left(0,\;\frac{1}{\alpha_{2}\cdots \alpha_{K-1}},\;\cdots ,\frac{1}{\alpha_{K-1}},\;1\right)$, ⋯, $\left(0,\;0,\;\cdots 0,\;\frac{1}{\alpha_{K-1}},1\right)$, (0, ⋯, 0, 1). The coordinate averages of these vectors result in the centralized utility assignments $\Delta u_{i}=\frac{i}{K}{\left(\prod_{j=i}^{K-1}\alpha_{j}\right)}^{-1}-\frac{i-1}{K}{\left(\prod_{j=i-1}^{K-1}\alpha_{j}\right)}^{-1}$ using $U_{i}=\frac{i}{K}{\left(\prod_{j=i}^{K-1}\alpha_{j}\right)}^{-1}$, i=1,⋯,K−1, $U_{K}=1$.

## Appendix B. (Interval Expressions of Utility Values)

### Appendix B.1. Interval Utility Values: $U_{IU}=\left\{LB_{i}\leq U_{i}\leq UB_{i},i=2,\cdots ,K-1\right\}$

In this case, each extreme point is determined by taking the lower and upper bounds of each $U_{i}$ alternately, and thus the total number of extreme points is $2^{K-2}$. To list them,

$$\left(0,\;LB_{2},\;LB_{3},\;\cdots ,\;LB_{K-1},\;1\right),\;\left(0,\;LB_{2},\;\cdots ,\;LB_{K-2},UB_{K-1},\;1\right),\cdots ,\left(0,\;UB_{2},\;UB_{3},\;\cdots ,\;UB_{K-1},\;1\right).$$

### Appendix B.2. Interval Differences of Utility Values: $U_{IDU}=\left\{LB_{i}\leq U_{i}-U_{i-1}\leq UB_{i},i=1,\cdots ,K\right\}$

To start with, we make the change of variables $\Delta u_{i}=U_{i}-U_{i-1}$, which transforms the original set of bounded differences into

$$\left\{LB_{i}\leq \Delta u_{i}\leq UB_{i},i=1,\;\cdots ,\;K,\;\sum_{i=1}^{K}\Delta u_{i}=1\right\}.$$

The extreme points are easily identified by taking at least K−1 lower or upper bounds of $\Delta u_{i}$ that sum to one. Suppose that $\sum_{i=1}^{K-1}LB_{i}+\alpha =1$, $\alpha \in \left[LB_{K},\;UB_{K}\right]$. Then we solve a set of equations to determine the extreme point in terms of $U_{i}$:

$$U_{1}-U_{0}=LB_{1},$$

$$U_{2}-U_{1}=LB_{2},$$

⋯

$$U_{K-1}-U_{K-2}=LB_{K-1},$$

$$U_{K}-U_{K-1}=\alpha .$$

The resulting extreme points will be $\left(0,\;LB_{1},\;LB_{1}+LB_{2},\cdots ,\sum_{i=1}^{K-1}LB_{i},\;1\right)$. To illustrate, suppose that with K=4 $\left(U_{0}=0,\;U_{3}=1\right)$,

$$\left\{0.3\leq U_{1}-U_{0}\leq 0.5,\;0.2\leq U_{2}-U_{1}\leq 0.3,\;0.1\leq U_{3}-U_{2}\leq 0.3\right\}.$$

By introducing $\Delta u_{i}=U_{i}-U_{i-1}$, i=1, 2, 3, we obtain

$$\left\{0.3\leq \Delta u_{1}\leq 0.5,\;0.2\leq \Delta u_{2}\leq 0.3,\;0.1\leq \Delta u_{3}\leq 0.3,\sum_{i=1}^{3}\Delta u_{i}=1\right\}.\;$$

Then, the extreme points are simply reduced to {(0.5, 0.2,0.3), (0.5, 0.3,0.2), (0.4, 0.3, 0.3)}. The first extreme point is determined by selecting three end points of $\Delta u_{i}$, and the last two by selecting two end points and one interior point lying between the lower and upper bounds of $\Delta u_{i}$ [25]. Now, we solve a set of equations to obtain the extreme point in terms of $U_{i}$ such as (0, 0.5, 0.7, 1),

$$U_{1}-U_{0}=0.5,\;U_{2}-U_{1}=0.2,\;U_{3}-U_{2}=0.3.$$

Similar computations give the other extreme points (0, 0.5, 0.8, 1) and (0, 0.4, 0.7, 1).

### Appendix B.3. Interval Ratios of Utility Values: $U_{IRU}=\left\{LB_{i}\leq U_{i}/U_{i-1}\leq UB_{i},i=2,\cdots ,K\right\}$

To illustrate, suppose without loss of generality that every judgment on $U_{i}$, i=1, 2, is made relative to the most preferred $U_{3}$:

$$2\leq U_{3}/U_{1}\leq 3,\;4\leq U_{3}/U_{2}\leq 5.$$

We can further identify a ratio $U_{2}/U_{1}$, say $\frac{2}{5}\leq U_{2}/U_{1}\leq \frac{3}{4}$ from the given interval ratios. Then, we denote $q_{1}=U_{2}/U_{1}$, $q_{2}=U_{3}/U_{2}$, and $q_{3}=U_{1}/U_{3}$ to obtain

$$Q=\left\{\frac{2}{5}\leq q_{1}\leq \frac{3}{4},\;4\leq q_{2}\leq 5,\;\frac{1}{3}\leq q_{3}\leq \frac{1}{2},\;q_{1}\cdot q_{2}\cdot q_{3}=1\right\}.$$

The extreme points of Q are determined as follows:

$$\left(\frac{2}{5},\;5,\frac{1}{2}\right),\;\left(\frac{3}{4},\;4,\frac{1}{3}\right),\;\left(\frac{3}{5},\;5,\frac{1}{3}\right),\;\left(\frac{1}{2},\;4,\;\frac{1}{2}\right).$$

To obtain the extreme points in terms of $U_{i}$, we solve a system of equations. For example, with respect to $\left(\frac{2}{5},\;5,\frac{1}{2}\right)$, we construct the following set of equations to obtain $\left(0,\;\frac{1}{2},\frac{1}{5},\;1\right)$

$$\frac{U_{2}}{U_{1}}=\frac{2}{5},\;\frac{U_{3}}{U_{2}}=5,\;\frac{U_{1}}{U_{3}}=\frac{1}{2}.$$

Similarly, we can find other extreme points such as

$$\left(0,\;\frac{1}{3},\frac{1}{4},\;1\right),\;\left(0,\;\frac{1}{3},\frac{1}{5},\;1\right),\;\left(0,\;\frac{1}{2},\frac{1}{4},\;1\right).$$
